# The impact of intradialytic cycling on temporal changes in muscle sodium during hemodialysis

**DOI:** 10.1093/ckj/sfag193

**Published:** 2026-07-15

**Authors:** Hsin-Yu Fang, Luis M Perez, Ryan J Larsen, Bradley P Sutton, Gwendolyn R Derk, Talat A Ikizler, Kenneth R Wilund

**Affiliations:** Department of Kinesiology and Community Health, University of Illinois Urbana-Champaign, Urbana, IL, USA; Division of Nutritional Sciences, University of Illinois Urbana-Champaign, Urbana, IL, USA; Department of Kinesiology and Community Health, University of Illinois Urbana-Champaign, Urbana, IL, USA; Beckman Institute, University of Illinois Urbana-Champaign, Urbana, IL, USA; Beckman Institute, University of Illinois Urbana-Champaign, Urbana, IL, USA; Department of Bioengineering, University of Illinois Urbana-Champaign, Urbana, IL, USA; Department of Kinesiology and Community Health, University of Illinois Urbana-Champaign, Urbana, IL, USA; Division of Nephrology, Vanderbilt University Medical Center, Nashville, TN, USA; Department of Kinesiology and Community Health, University of Illinois Urbana-Champaign, Urbana, IL, USA; Division of Nutritional Sciences, University of Illinois Urbana-Champaign, Urbana, IL, USA

**Keywords:** end stage kidney disease, hemodialysis, intradialytic exercise, muscle sodium kinetics, sodium magnetic resonance imaging

## Abstract

**Background:**

Sodium magnetic resonance imaging (^23^Na-MRI) studies indicate that patients undergoing maintenance hemodialysis (MHD) exhibit elevated muscle sodium concentrations that are associated with deleterious consequences. While muscle sodium concentrations have been shown to decline during a hemodialysis (HD) session, little is known about whether they subsequently rebound or how intradialytic muscle sodium dynamics might be modulated. We thus examined muscle sodium kinetics on HD and non-HD days and evaluated if an acute intradialytic exercise bout could enhance HD-associated changes in muscle sodium concentration.

**Methods:**

Seven MHD patients participated in this pilot consisting of two studies. The ‘Time-Series’ study included four sequential ^23^Na-MRI scans between two HD treatments at: (1) pre-HD1 (T1); (2) post-HD1 (T2); (3) 24 h post-HD1 (T3); and 4) pre-HD2 (T4). The ‘Na-Exercise’ study included two ^23^Na-MRI scans: (1) a pre-HD scan (T5); and (2) a post-HD (T6) scan on a day where participants cycled for 30 min during HD. Calf muscle sodium concentrations were quantified from ^23^Na-MRI images.

**Results:**

After decreasing during HD (*P* < .001), calf muscle sodium concentration returned to the pre-HD1 level by 24 h post-HD1 (*P* = .84) and was numerically, but not significantly, lower than baseline at pre-HD2 (*P* = .07). The intradialytic cycling bout did not significantly affect the decline in calf muscle sodium concentrations during HD (group × time interaction: *P* = .13).

**Conclusions:**

Calf muscle sodium concentrations decrease during HD, return to baseline within 24 h, and may show a smaller decline before the start of the next session. The effect of a single intradialytic cycling bout on the intradialytic reduction in muscle sodium concentration was not clearly detectable and remains inconclusive.

## Graphical Abstract

**Graphical Abstract:**
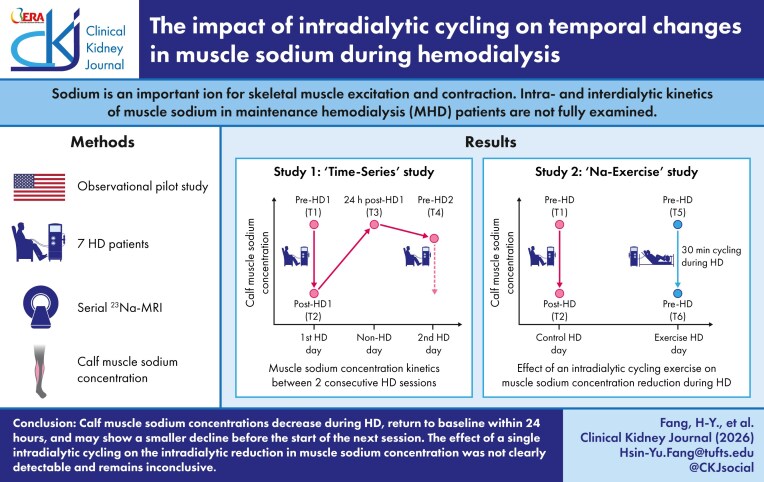

KEY LEARNING POINTS **What was known:**

- Muscle sodium concentration determined by lower leg ^23^Na-MRI is higher in patients on MHD than in their healthy counterparts.
- Greater muscle sodium accumulation in MHD patients is associated with muscle insulin resistance and inflammation.
- A single HD session leads to an acute reduction in muscle sodium concentration determined by lower leg ^23^Na-MRI.

**This study adds:**

- Immediately after an HD treatment, muscle sodium concentration in MHD patients is reduced but returns to the pre–HD level by 24 h, followed by a potential post–rebound drop before the next session.
- The reduction in muscle sodium concentration during HD does not appear to be influenced by an acute 30-min bout of intradialytic cycling exercise, but additional studies are needed to clarify this effect.
- There is no correlation observed between intradialytic muscle sodium concentration changes and ultrafiltration volume or dialysate-to-plasma sodium gradient.

**Potential impact:**

- This study offers new insights into muscle sodium accumulation in MHD patients by describing intra- and interdialytic muscle sodium kinetics.
- Our observation of a rapid post–HD rebound in muscle sodium concentration, followed by a potential post–rebound decline before the next session, suggests a highly dynamic and complex equilibrium of tissue sodium after HD.
- This study also provides pilot data on the effect of a single acute bout of intradialytic cycling on intradialytic muscle sodium concentration reduction, helping to guide future larger trials on whether exercise can modify muscle sodium kinetics and clearance in MHD patients.

## INTRODUCTION

Sodium represents an important ion involved in skeletal muscle excitation and contraction. Under healthy conditions, the intracellular sodium concentration ([Na^+^]_i_) in resting human skeletal muscle is 5–26 Mm [1–3], much lower than the extracellular sodium concentration ([Na^+^]_o_) of about 140 mM [4–6]. This sodium concentration gradient across the sarcolemma is maintained by sodium-potassium pump (Na^+^-K^+^-ATPase) [6] and regulates muscle excitability with other factors [7,8]. Diminished trans-sarcolemmal sodium gradients resulting from increased [Na^+^]_i_ (experimentally equivalent to decreased [Na^+^]_o_) and/or insufficient Na^+^-K^+^-ATPase activity impede action potential propagation and consequently muscle contractility [7–10]. In patients with end-stage kidney disease (ESKD), the skeletal muscle exhibits elevated [Na^+^]_i_ [11] and impaired Na^+^-K^+^-ATPase activity [12], whereby a normal trans-sarcolemmal sodium gradient might not be established. These abnormalities may partly explain skeletal muscle dysfunction commonly observed in patients with advanced kidney disease.

Non-invasive *in-vivo* measurements of sodium in human skeletal muscle are enabled by sodium magnetic resonance imaging (^23^Na-MRI) [13]. Recent ^23^Na-MRI studies suggest that in patients with ESKD on maintenance hemodialysis (MHD), muscle sodium accumulation, besides its potential role in muscle dysfunction mentioned above, is linked with muscle insulin resistance [14] and other complications [15]. It remains unknown if muscle sodium can be manipulated to prevent its associated complications, with no established interventions to reduce muscle sodium. While a single HD session has been demonstrated to acutely reduce calf muscle sodium concentration in MHD patients [16,17], it is unclear whether this reduction sustains over time. Therefore, the primary aim of this study was to characterize temporal changes in calf muscle sodium concentrations across two consecutive HD sessions in MHD patients using ^23^Na–MRI. We hypothesized that muscle sodium concentrations would decline immediately after an HD session relative to pre–HD values, followed by a yet undefined post–HD response occurring before the subsequent HD treatment.

Intradialytic cycling exercise has been reported to enhance the dialytic clearance of urea [18,19], phosphate [20,21], and potassium [19] in patients on MHD, likely through increased blood flow to exercising muscles that facilitates tissue solute efflux into the intravascular compartment, where these solutes are subsequently removed by HD [19,22]. Thus, the secondary aim of this study was to evaluate whether an acute bout of intradialytic cycling exercise could augment the reduction in calf muscle sodium concentration observed immediately post–HD compared with pre–HD values.

## MATERIALS AND METHODS

### Patient population

MHD patients were recruited from an outpatient HD clinic in Champaign, Illinois between August and December 2019. Major inclusion criteria were MHD patients who were older than 18 years and had been on thrice-weekly HD for a minimum of 3 months. Exclusion criteria were contraindications to MRI, lack of physician clearance for exercise, and plans to begin or ongoing participation in dietary interventions at the time of the study. The study was approved by the Institutional Review Board at the University of Illinois Urbana-Champaign (Protocol #16417) and was conducted complying with the Declaration of Helsinki.

### Study set-up

**Figure 1:**
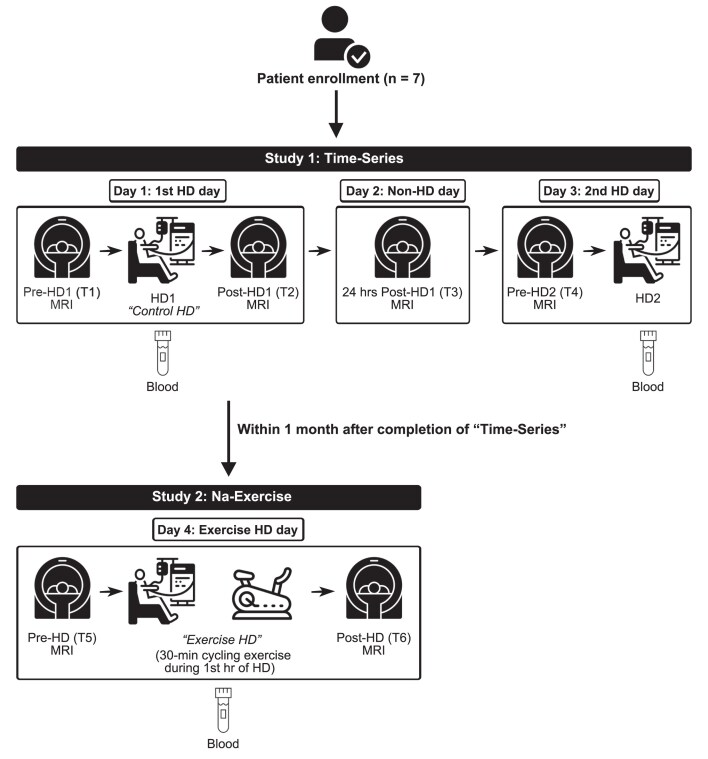
Research design. All participants participated in both studies, which took 4 days in total. The first “Time-Series” study consisted of two consecutive HD days (Day 1 and 3) and the non-HD day in between (Day 2), aiming to investigate calf muscle sodium kinetics between HD treatments. During this study, patients received ^23^Na-MRI to determine calf muscle sodium concentration at four time points: pre-HD1 (T1), post-HD1 (T2), 24 h post-HD1 (T3), and pre-HD2 (T4). Within a month after completion of “Time-Series,” all patients participated in the “Na-Exercise” study, where they underwent a pair of pre- and post-HD ^23^Na-MRI scans (at T5 and T6) on an Exercise HD day (Day 4) in which they cycled for 30 min on an ergometer during HD. The effect of this intradialytic exercise bout on HD-enforced muscle sodium concentration reduction was evaluated by comparing T5–T6 and T1–T2 changes in calf muscle sodium concentration. The HD sessions on Day 1 and Day 4 served as the control and exercise HD sessions, respectively. Blood was collected at the start and the end of HD on Day 1, 3, and 4.

The research design (Fig. 1) included two studies to examine: (1) temporal changes in calf muscle sodium concentrations between two consecutive HD sessions; and (2) the effect of an acute bout of intradialytic cycling on the reduction in calf muscle sodium concentration during HD, respectively. In the first study (named ‘Time-Series’), all participants underwent four ^23^Na-MRI scans over three consecutive days, including two HD days and the non-HD day in between. Scans were conducted at four timepoints: (1) pre-HD1 (T1); (2) post-HD1 (T2); (3) 24 hours post-HD1 (T3); and (4) pre-HD2 (T4).

Within 1 month (mean duration: 15.7 ± 8.3 days) after completing the Time-Series study, all participants joined the second study named ‘Na-Exercise.’ This second study required participants to undergo two additional ^23^Na-MRI scans, one immediately before and one immediately after an HD session (at T5 and T6, respectively) in which they cycled for 30 min during the first hour of HD. The intradialytic cycling session was performed supervised on a Monark 871E ergometer (Monark Exercise, Sweden) at intensities ranging from ‘very light’ (warm-up/cool-down) to ‘somewhat hard’ (during most of the session), corresponding to a range of 9–13 on the Borg Scale of Perceived Exertion [23]. The effect of intradialytic cycling on the intradialytic reduction in calf muscle sodium concentration was assessed by comparing the changes in calf muscle sodium concentrations during the control (T1–T2) and exercise (T5–T6) HD sessions.

We conducted the Time-Series study before Na-Exercise without randomizing their order to ensure we captured data for the primary aim, even if participants dropped out between studies. Had we reversed the order, early dropouts would have left us with only the known acute effect of HD on muscle sodium [16,17]. Patients were instructed to maintain their usual diets throughout the study.

### ^23^Na-MRI and calf muscle sodium quantification

Calf muscle sodium concentration in the right leg was measured using a 3T Prisma MRI scanner (Siemens, Erlangen, Germany) with custom ^1^H and ^23^Na coils, following a previous protocol [24]. The leg was positioned in both coils with three NaCl aqueous solution standards (10, 30, 50 mM), and leg placement reproducibility was ensured by measuring leg protrusion. T1-weighted ^1^H imaging was conducted using a FLASH sequence (TR/TE = 7.5/3.69 ms, 5° flip angle, FOV of 280 × 280 mm, a matrix size of 256 × 256, 16 slices at a thickness of 5 mm with no gap, 8 averages for a scan time of 4.1 min). ^23^Na imaging was performed using the flexTPI sequence [23] (TR/TE = 200/0.5 ms, 70° flip angle, FOV = 280 × 280 × 280 mm, effective matrix size of 44 × 44 × 44, three averages, total scan time of 15.8 min). Data were reconstructed on a 64 × 64 × 64 matrix using a gridding algorithm on MATLAB (R2018b; MathWorks Inc., MA) [25] and analyzed to quantify calf muscle sodium concentration using ImageJ (NIH, version 1.52a): regions-of-interest (ROIs) were drawn over the soleus and both gastrocnemius heads on the ^1^H image, applied to the ^23^Na image, and ^23^Na signal intensities were converted to concentrations via a fitted calibration curve. Calf muscle sodium concentration was calculated by averaging the measured sodium concentrations in the three ROIs.

### Blood chemistry and dialysis parameters

Blood samples were collected from participants [24] within 5 min of starting (pre-heparin) and ending HD, and analyzed for plasma metabolites and electrolytes using a Piccolo-Xpress Chemistry Analyzer (Abaxis, CA). HD parameters were recorded from medical records.

### Body fluid volumes, body weight, and blood pressure

Fluid volume parameters, including total body water (TBW), extracellular fluid (ECF), and intracellular fluid (ICF), were assessed using bioimpedance spectroscopy (ImpediMed SFB7, Carlsbad, CA, USA). Body weight was recorded at the time of the bioimpedance measurement. Systolic and diastolic blood pressure (SBP/DBP) and mean arterial pressure (MAP) were measured after 5 min of seated rest using an automated oscillometric cuff (Mobil–O–Graph, IEM, Stolberg, Germany).

### Sample size calculation

Sample size was estimated using GPower 3.1 software based on a prior study showing a 4.6 ± 3.6 mM reduction in muscle sodium after HD in non-diabetic MHD patients [17]. To detect a similar effect with 80% power at a 0.05 one-sided significance level, at least six participants were needed. No sample size calculation was done for the secondary aim due to limited prior research investigating exercise effects on muscle sodium in MHD patients.

### Statistics

Calf muscle sodium concentration changes over time (primary outcome) were assessed using linear mixed models in RStudio (lme4 package, v3.6.1). To assess intra- and interdialytic sodium kinetics, ‘Time-Series’ data were modeled with a fixed time effect and a random participant intercept, and resultant β-coefficients reflected changes at post-HD timepoints (T2–T4) relative to baseline (T1). To evaluate the impact of the intradialytic cycling bout on intradialytic muscle sodium concentration reduction, muscle sodium data from control (T1–T2) and exercise HD (T5–T6) sessions were compared using a linear mixed model with fixed effects for group (exercise HD vs. control HD), time (pre-HD vs. post-HD), and their interaction. The interaction-term β coefficient quantified the difference in intradialytic muscle sodium concentration change between the control and exercise sessions. The same approach was applied to plasma sodium. Pooled data from both HD sessions were analyzed using repeated-measures correlation (rmcorr R package [25]) to assess associations between intradialytic muscle sodium concentration changes, plasma sodium concentration changes, and the pre-HD dialysate-to-plasma sodium gradient. Differences in pre- or post-HD outcomes between control and exercise HD were tested using paired *t*-test or sign test (SPSS Statistics version 26, IBM, Chicago, IL). Significance was set at two-tailed *P* < .05, and data are reported as mean ± SD or counts with percentages.

## RESULTS

### Patient characteristics

Seven patients undergoing thrice-weekly MHD (age = 60 ± 13 years; BMI = 36 ± 10 kg/m^2^) participated in both ‘Time-Series’ and ‘Na-Exercise’ studies. Table 1 displays patient demographics, primary cause of kidney failure, comorbidities, and medication use. To align with the biomedical imaging center’s schedule, enrollment was limited to patients on the second dialysis shift.

**Table 1:** Patient characteristics.

Characteristics	HD patients (*n* = 7)
Age, yr, mean ± SD	60 ± 13
Male sex, *n* (%)	4 (57)
BMI, kg/m^2^, mean ± SD	36 ± 10
Ethnicity, *n* (%)	
White	3 (43)
African American	4 (57)
Primary cause of renal failure, *n* (%)	
Diabetes mellitus	3 (43)
Hypertension	4 (57)
Co-morbidities, *n* (%)	
Hypertension	6 (86)
Diabetes mellitus	5 (71)
History of cardiovascular disease	5 (71)
Secondary hyperparathyroidism	7 (100)
Hyperlipidemia	3 (43)
Anemia	7 (100)
Obesity (BMI > 30 kg/m^2^)	5 (71)
Medication use, *n* (%)	
Antihypertensive agents	
ACEIs/ARBs	7 (100)
Beta-blockers	1 (14)
Alpha-blockers	5 (71)
Combined alpha- and beta-blockers	2 (29)
Calcium channel blockers	5 (71)
Central agonists	4 (57)
Vasodilators	3 (43)
Diuretics	3 (43)
NSAIDs	6 (86)
Statins	5 (71)
Phosphate binders	6 (86)
Calcimimetics	4 (57)
Erythropoiesis-stimulating agents	6 (86)

Values are mean ± SD or number (percentage).

Abbreviations: BMI, body mass index; ACEs, angiotensin-converting enzyme inhibitors; ARBs, angiotensin receptor blockers; NSAIDs, nonsteroidal anti-inflammatory drugs.

### Blood chemistry and dialysis parameters

Table 2 summarizes blood chemistry and dialysis parameters from control and exercise HD sessions, revealing no significant differences in plasma analytes or dialysis metrics pre- and post-HD. Dialysate prescriptions remained unchanged throughout the study.

**Table 2:** Comparisons of blood chemistry and dialysis parameters between control and exercise HD sessions.

		Control HD^a^ (*n* = 7)	Exercise HD^b^(*n* = 7)	*P* value
Blood metabolites and electrolytes				
Glucose, mg/dl	Pre-HD	122 ± 21	141 ± 48	.24
	Post-HD	114 ± 31	106 ± 18	.34
BUN, mg/dl	Pre-HD	50 ± 15	49 ± 18	.86
	Post-HD	14 ± 6^d^	13 ± 6^d^	.67
Calcium, mg/dl	Pre-HD	9.4 ± 0.9	9.2 ± 0.9	.22
	Post-HD	9.0 ± 0.4	8.9 ± 0.3	.43
Creatinine, mg/dl	Pre-HD	8.3 ± 3.2	8.6 ± 3.4	.19
	Post-HD	2.8 ± 0.8^e^	2.9 ± 1.2^f^	.64
Albumin, g/dl	Pre-HD	3.2 ± 0.2	3.1 ± 0.3	.22
	Post-HD	3.4 ± 0.3	3.4 ± 0.2^d^	.90
Phosphorus, mg/dl	Pre-HD	6.0 ± 1.5	6.0 ± 0.7	.97
	Post-HD	3.0 ± 0.5^e^	2.7 ± 0.4^f^	.69
Sodium, mmol/l	Pre-HD	140.9 ± 1.8	141.4 ± 1.9	.55
	Post-HD	138.4 ± 3.4	139.3 ± 3.3	.56
Potassium, mmol/l	Pre-HD	5.1 ± 0.7	5.2 ± 0.7	.20
	Post-HD	3.4 ± 0.3^d^	3.5 ± 0.5^f^	.99
Chloride, mmol/l	Pre-HD	101.4 ± 2.2	101.6 ± 4.1	.73
	Post-HD	99.4 ± 1.5^d^	99.6 ± 2.4	.99
Dialysis parameters				
Average BFR, ml/min		406 ± 67	430 ± 54	.47
Average DFR, ml/min		647 ± 89	681 ± 106	.48
Blood volume processed, l		88 ± 17	88 ± 13	.98
spKt/V		1.54 ± 0.32	1.46 ± 0.25	.99
Total UF volume, ml		3095 ± 1072	2919 ± 700	.73
HD treatment time, minutes		233 ± 15	229 ± 15	.55
Estimated dry weight, kg		100.6 ± 29.1	100.1 ± 29.2	.36
Dialysate potassium, mEq/l^c^		2 ± 0	2 ± 0	─
Dialysate calcium, mEq/l^c^		3 ± 0	3 ± 0	─
Dialysate magnesium, mEq/l^c^		1 ± 0	1 ± 0	─
Dialysate sodium, mEq/l^c^		136 ± 2	136 ± 2	─
Dialysate bicarbonate, mEq/l^c^		34 ± 2	34 ± 2	─
Dialysate dextrose, mg/dl^c^		100 ± 0	100 ± 0	─

Values are shown as mean ± SD.

a The first HD session in the ‘Time-Series’ study.

b The HD session in the ‘Na-Exercise’ study.

c Prescribed dialysate composition remained the same for each patient throughout the entire study period, and hence a paired *t*-test was not applicable.

d Post-HD values significantly different from pre-HD values (*P* < .05, paired *t*- or sign test).

^e^Post-HD values significantly different from pre-HD values (*P* < .01, paired *t*- or sign test).

^f^Post-HD values significantly different from pre-HD values (*P* < .001, paired *t*- or sign test).

Abbreviations: HD, hemodialysis; BUN, blood urea nitrogen; BFR, blood flow rate; DFR, dialysate flow rate; spKt/V, single-pool Kt/V; UF, ultrafiltration.

### Body fluid volumes, body weight, and blood pressure indices

Table 3 compares fluid volume parameters (i.e. TBW, ECF, and ICF), body weight, and hemodynamic indices (i.e. SBP, DBP, and MAP) measured at pre- and post-HD during both control and exercise sessions. All measured parameters were comparable between control and exercise sessions at both pre- and post-HD timepoints (*P* > .05). Linear mixed modeling revealed no significant group × times interactions, suggesting that intradialytic changes were comparable across both HD sessions for all parameters. Furthermore, a significant main effect of time was observed for all parameters except ICF (*P* = .06), indicating a general decrease from pre- to post-HD regardless of the HD session condition. No significant main effect of group was found for any variable.

**Table 3:** Comparisons of intradialytic changes in body fluid volumes, body weight, and blood pressure indices between control and exercise HD sessions (linear mixed model analysis).

	Control HD (*n* = 7)			Exercise HD (*n* = 7)			Linear mixed model analysis^a^			
							*P* value			
	Pre-HD^b^	Post-HD^c^	∆post-pre (%change)	Pre-HD^d^	Post-HD^e^	∆post-pre (%change)	Group effect	Time effect	Group x Time effect	R^2^_m_/R^2^_c_
Body fluid volumes and body weight										
TBW, L	52.4 ± 10.3	48.5 ± 11.2	−3.92 (−7.5%)	51.0 ± 12.6	48.6 ± 12.4	−2.32 (−4.6%)	.43	<.001	.33	0.02/0.96
ECF, L	22.7 ± 5.8	22.0 ± 5.4	−0.74 (−3.3%)	23.5 ± 6.7	21.5 ± 5.2	−1.96 (−8.4%)	.74	.03	.31	0.02/0.92
ICF, L	29.6 ± 5.7	26.5 ± 7.2	−3.13 (−10.5%)	27.5 ± 6.5	27.1 ± 7.5	−0.36 (−1.3%)	.37	.06	.13	0.04/0.86
BW, kg	103.7 ± 29.2	100.8 ± 29.2	−2.90 (−2.8%)	103.5 ± 30.3	100.7 ± 30.1	−2.76 (−2.7%)	.83	<.001	.91	0.003/> 0.99
Blood pressure indices										
SBP, mmHg	168 ± 24	150 ± 31	−17.9 (−10.7%)	164 ± 23	153 ± 25	−11.7 (−7.1%)	.96	.004	.50	0.09/0.78
DBP, mmHg	98 ± 18	89 ± 16	−9.0 (−9.2%)	96 ± 18	86 ± 20	−9.3 (−9.7%)	.43	.009	.96	0.08/0.76
MAP, mmHg	128 ± 16	115 ± 19	−13.2 (−10.3%)	125 ± 17	115 ± 19	−10.3 (−8.2%)	.66	.003	.68	0.12/0.72

Values are shown as mean ± SD. R^2^_m_ and R^2^_c_ represent the proportion of variance explained by solely the fixed effect and by the full model, respectively. R^2^_m_, marginal R^2^; R^2^_c_, conditional R^2^.

a The linear mixed model used included fixed-effect terms for group (control vs. exercise), time (pre-HD vs. post-HD), the group × time interaction, and a random participant intercept.

b Timepoint T1 in the ‘Time-Series’ study.

c Timepoint T2 in the ‘Time-Series’ study.

d Timepoint T5 in the ‘Na-Exercise’ study.

e Timepoint T6 in the ‘Na-Exercise’ study.

Abbreviations: TBW, total body water; ECF, extracellular fluid; ICF, intracellular fluid; BW, body weight; SBP, systolic blood pressure; DBP, diastolic blood pressure; MAP, mean arterial pressure.

Table 4 presents the body fluid volumes, body weight, and hemodynamic indices measured at four timepoints between the two consecutive HD sessions in the ‘Time-Series’ study. Linear mixed modeling revealed a significant main effect of time for body weight and all three blood pressure indices; however, the main effect of time was non-significant for all fluid volume parameters. Post–hoc pairwise comparisons showed that, compared with measurements at T1, body weight was decreased at T2 through T4, and all three blood pressure indices were decreased at T2.

**Table 4:** Comparisons of body fluid volumes, body weight, and blood pressure indices at each timepoint between two consecutive HD sessions (linear mixed model analysis).

Variables	T1	T2	T3	T4	*P* value (T2 vs. T1)	*P* value (T3 vs. T1)	*P* value (T4 vs. T1)	*P* value (time effect)
Body fluid volumes and body weight								
TBW, L	52.4 ± 10.3	48.5 ± 11.2	52.1 ± 12.3	50.2 ± 13.4	.03	.87	.20	.10
ECF, L	22.7 ± 5.8	22.0 ± 5.4	23.2 ± 5.8	22.9 ± 5.4	.53	.67	.85	.74
ICF, L	29.6 ± 5.7	26.5 ± 7.2	28.9 ± 7.0	27.7 ± 8.6	.07	.65	.24	.27
BW, kg	103.7 ± 29.2	100.8 ± 29.2	101.9 ± 29.1	102.6 ± 29.7	<.001	<.001	.01	<.001
Blood pressure indices								
SBP, mmHg	168 ± 24	150 ± 31	160 ± 31	165 ± 20	<.01	.20	.70	.03
DBP, mmHg	98 ± 18	89 ± 16	95 ± 17	98 ± 14	<.01	.32	.89	.03
MAP, mmHg	128 ± 16	115 ± 19	123 ± 18	129 ± 12	<.01	.17	.94	<.01

Values are shown as mean ± SD.

*P* values were derived by a linear mixed model including a fixed-effect term of time (T1–T4) and a random participant intercept.

Abbreviations: T1, pre-HD1; T2, post-HD1; T3, 24 h post-HD1; T4, pre-HD2; TBW, total body water; ECF, extracellular fluid; ICF, intracellular fluid; BW, body weight; SBP, systolic blood pressure; DBP, diastolic blood pressure; MAP, mean arterial pressure.

### Calf muscle sodium kinetics between two consecutive HD sessions

**Figure 2:**
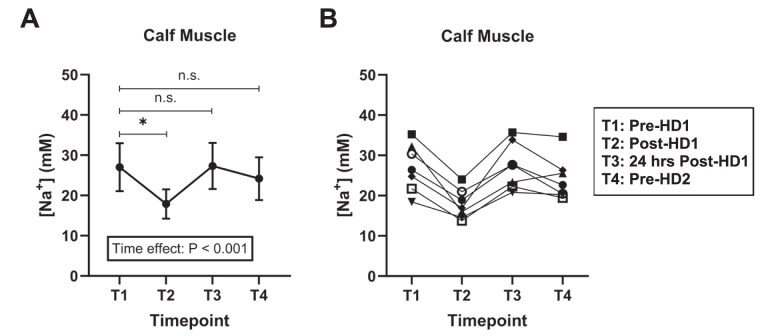
Calf muscle sodium concentrations at different timepoints between two consecutive HD sessions. (A) Summary data. (B) Individual data (*n* = 7). The summary data plot displays the means and SDs of the calf muscle sodium concentrations at timepoints T1–T4: T1, 27.0 ± 6.0 mM; T2, 17.9 ± 3.7 mM; T3, 27.3 ± 5.7 mM; T4, 24.2 ± 5.3 mM. Linear mixed model analysis showed a significant time effect for calf muscle sodium concentrations over T1–T4 (*P* < .001). The analysis further showed that T2 was the only timepoint at which calf muscle sodium concentration deviated from the value at T1: *P < .001; n.s., non-significant. HD, hemodialysis; [Na^+^], sodium concentration.

**Figure 3:**
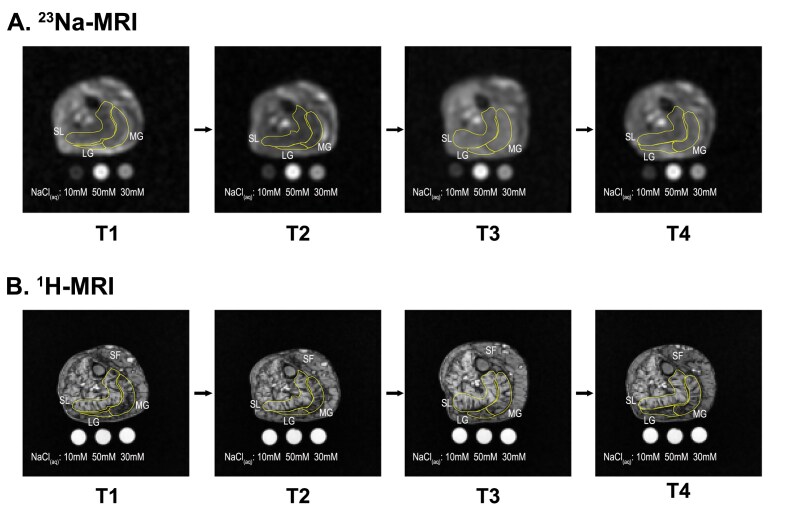
Representative (A) ^23^Na-MRI and (B) ^1^H-MRI images for timepoints T1–T4 obtained from the same patient. The calf muscle sodium concentration at each timepoint was derived by averaging the sodium concentrations in the three component muscles (outlined areas) which were quantified by referencing the ^23^Na signals in muscles to those in the 10, 30, and 50 mM NaCl standards: T1, 21.7 mM; T2, 13.8 mM; T3, 22.3 mM; T4, 19.5 mM. Muscle boundaries were defined on the ^1^H-MRI images and then applied to corresponding ^23^Na-MRI images. SL, soleus; MG, medial gastrocnemius; LG, lateral gastrocnemius; SF, subcutaneous fat; NaCl_(aq)_, sodium chloride aqueous solution.

Figure 2 presents calf muscle sodium concentrations at four timepoints during the ‘Time-Series’ study: T1, 27.0 ± 6.0 mM; T2, 17.9 ± 3.7 mM; T3, 27.3 ± 5.7 mM; T4, 24.2 ± 5.3 mM. Corresponding representative ^23^Na- and ^1^H-MRI images are shown in Fig. 3A and B, respectively. Linear mixed model analysis (Table 5, Model 1) showed that, compared with T1, calf muscle sodium concentration decreased at T2 (β = −9.15 mM, *P* < .001), returned to baseline at T3 (β = 0.30 mM, *P* = .84), and was numerically but not significantly lower than baseline at T4 (β = −2.85 mM, *P* = .07). This result persisted after adjustment for fluid volume parameters (i.e. TBW, ECF, ICF) and body weight, which were included as time–varying covariates (Table 5, Model 2–5).

**Table 5:** Changes (∆) in calf muscle sodium concentration over time between two consecutive HD sessions (linear mixed model analysis).

Calf muscle sodium concentration, mM (*n* = 7)									
	∆T2 − T1		∆T3 − T1		∆T4 − T1				
Model	β (95% CI)	*P* value	β (95% CI)	*P* value	B (95% CI)	*P* value	Time effect P value	R^2^_m_/R^2^_c_	AIC
Model 1(Time)	−9.15(−12.16, −6.14)	<.001	0.30(−2.71, 3.31)	.84	−2.85(−5.86, 0.16)	.07	<.001	0.39/0.80	163.7
Model 2(Time + TBW)	−9.01(−12.14, −5.86)	<.001	0.31(−2.70, 3.32)	.83	−2.77(−5.82, 0.29)	.08	<.001	0.39/0.80	165.6
Model 3(Time + ECF)	−9.08(−12.11, −6.04)	<.001	0.26(−2.78, 3.28)	.86	−2.87(−5.90, 0.15)	.06	<.001	0.40/0.80	165.5
Model 4(Time + ICF)	−9.11(−12.27, −5.95)	<.001	0.31(−2.71, 3.33)	.83	−2.83(−5.90, 0.24)	.07	<.001	0.39/0.80	165.7
Model 5(Time + BW)	−9.38(−12.39, −6.37)	<.001	0.15(−2.84, 3.16)	.92	−2.94(−5.94, 0.06)	.06	<.001	0.51/0.81	163.5

Calf muscle sodium concentrations measured in the ‘Time-Series’ study were analyzed with the following linear mixed models:

Model 1: the model included a fixed-effect term of time (T1–T4) and a random participant intercept.

Model 2: Model 1 plus volume of total body water (TBW) as a fixed-effect term.

Model 3: Model 1 plus volume of extracellular fluid (ECF) as a fixed-effect term.

Model 4: Model 1 plus volume of intracellular fluid (ICF) as a fixed-effect term.

Model 5: Model 1 plus body weight (BW) as a fixed-effect term.

β coefficient estimates represent the average changes in calf muscle sodium concentration at the post-HD timepoints (T2, T3, and T4) relative to the pre-HD baseline (T1) in the ‘Time-Series’ study. R^2^_m_ and R^2^_c_ represent the proportion of variance explained by solely the fixed effect and by the full model, respectively. AIC is a model selection criterion, with a lower value indicating a better model fit.

Abbreviations: T1, pre-HD1; T2, post-HD1; T3, 24 hours post-HD1; T4, pre-HD2; R^2^_m_, marginal R^2^; R^2^_c_, conditional R^2^; AIC, Akaike information criterion.

**Figure 4:**
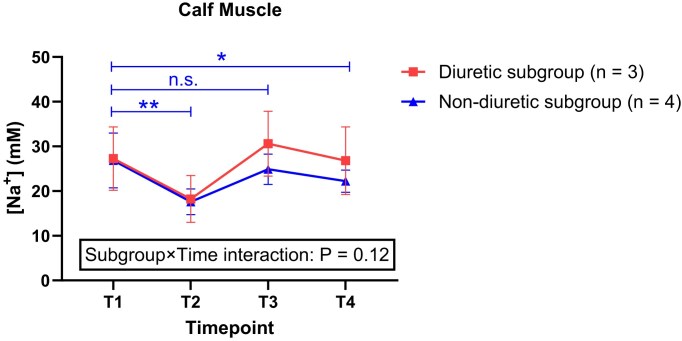
Calf muscle sodium concentrations at different timepoints between two consecutive HD sessions in diuretic and non-diuretic subgroups. The calf muscle sodium concentrations (mean ± SD) at time points T1–T4 for each subgroup: diuretic subgroup (n = 3): T1, 27.3 ± 7.1 mM; T2, 18.2 ± 5.2 mM; T3, 30.6 ± 7.3 mM; T4, 26.8 ± 7.6 mM; non–diuretic subgroup (n = 4): T1, 26.9 ± 6.1 mM; T2, 17.6 ± 2.9 mM; T3, 24.9 ± 3.4 mM; T4, 22.2 ± 2.5 mM. Linear mixed–model analysis showed a significant time effect for calf muscle sodium concentrations across T1–T4 (*P* < .001), with no significant subgroup effect or subgroup × time interaction. The analysis further showed that in the non-diuretic referent subgroup, calf muscle sodium concentration decreased at T2 compared with T1, rebounded toward baseline at T3, and was lower than baseline again at T4: *P < .01; **P < .001; n.s., non-significant. HD, hemodialysis; [Na^+^], sodium concentration.

Table 6 presents results from an additional linear mixed model for calf muscle sodium concentration, in which diuretic–use subgroups (use vs. non-use) and their interaction with time were included as fixed–effect terms, in addition to time. A significant main effect of time was observed (*P* < .001). Post–hoc pairwise comparisons indicated that within the non–diuretic reference subgroup, calf muscle sodium concentration decreased at T2 compared with T1 (β = −9.25 mM, *P* < .001), rebounded toward baseline at T3 (β = −1.98 mM, *P* = .25), and was lower than baseline again at T4 (β = −4.64 mM, *P* = .01). No significant main effect of subgroup was found (*P* = .38), and baseline concentrations at T1 were similar between subgroups (β = 0.40 mM, *P* = .91), suggesting that calf muscle sodium concentrations overall did not differ by diuretic status throughout T1–T4. The subgroup × time interaction was non-significant (*P* = .12); however, a borderline interaction at T3 (β = 5.33 mM, *P* = .05) suggested a numerically greater rebound in muscle sodium concentration within the diuretic subgroup relative to the non-diuretic subgroup. Calf muscle sodium concentration trajectories for both subgroups are shown in Fig. 4.

**Table 6:** Changes (∆) in calf muscle sodium concentration over time between two consecutive HD sessions, adjusted for diuretics use subgroups (linear mixed model analysis).

	β Estimate (95% CI)	*P* value
Time		<.001
T2 vs. T1	−9.25 (−12.72, −5.79)	<.001
T3 vs. T1	−1.98 (−5.45, 1.48)	.25
T4 vs. T1	−4.64 (−8.11, −1.17)	.01
Subgroup		.38
Diuretic vs. non-diuretic	0.40 (−7.04, 7.83)	.91
Time × subgroup interaction		.12
T2 × Diuretic	0.24 (−5.05, 5.54)	.93
T3 × Diuretic	5.33 (0.03, 10.63)	.05
T4 × Diuretic	4.18 (−1.12, 9.47)	.12

Calf muscle sodium concentrations measured in the ‘Time-Series’ study were analyzed using a linear mixed-effects model that included fixed effects for time (T1–T4), subgroup (diuretics use vs. non-use), the time × subgroup interaction, and a random intercept for each participant.

The β estimates for time represent the mean change in calf muscle sodium concentration at post-HD time points (T2, T3, and T4) relative to the pre-HD baseline (T1) within the non-diuretic subgroup.

The β estimate for subgroup represents the mean difference in calf muscle sodium concentration between the diuretic and non-diuretic subgroups at T1.

The β estimates for the time × subgroup interaction represent the additional change in calf muscle sodium concentration at each post-HD time point relative to T1 in the diuretic subgroup compared with the corresponding change in the non-diuretic subgroup (i.e. ∆T_i_ − T_1_*_Diuretic_*—∆T_i_ − T_1_*_Non-Diuretic_*; i = 2, 3, 4).

Abbreviations: T1, pre-HD1; T2, post-HD1; T3, 24 hours post-HD1; T4, pre-HD2.

### Plasma sodium kinetics between two consecutive HD sessions

**Figure 5:**
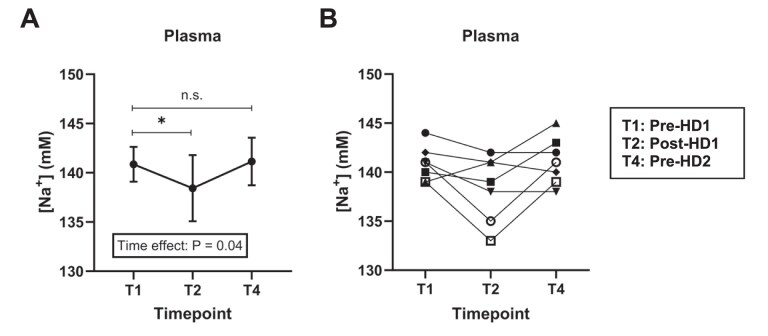
Plasma sodium concentrations at different timepoints between two consecutive HD sessions. (A) Summary data. (B) Individual data (*n* = 7). Plasma sodium concentration was only assessed at T1, T2, and T4. The summary data plot displays the means and SDs of the plasma sodium concentrations at each timepoint: T1, 141 ± 2 mM; T2, 138 ± 3 mM; T4, 141 ± 2 mM. Linear mixed model analysis showed a significant time effect for plasma sodium concentration over the course of T1, T2, and T4 (*P* = .04). The analysis also showed a decrease in plasma sodium concentration from T1 to T2, followed by a full return at T4: *P < .05, n.s., non-significant. HD, hemodialysis; [Na^+^], sodium concentration.

Figure 5 displays plasma sodium concentrations at T1 (140.9 ± 1.8 mM), T2 (138.4 ± 3.4 mM), and T4 (141.1 ± 2.4 mM) between consecutive HD sessions in ‘Time-Series’; T3 data were unavailable. Linear mixed model analysis (Table 7) showed a significant decrease in plasma sodium concentration at T2 compared to T1 (β = −2.43 mM, *P* = .04), with full recovery by T4 (β = 0.29 mM, *P* = .79).

**Table 7:** Changes (∆) in plasma sodium concentration over time between 2 consecutive HD sessions (linear mixed model analysis).

Plasma sodium concentration, mM(n = 7)			
	β (95% CI)	P value	R^2^_m_/R^2^_c_
Time effect		0.04	0.21/0.47
∆T2 − T1	−2.43 (−4.65, −0.20)	0.04	
∆T4 − T1	0.29 (−1.94, 2.51)	0.79	

The linear mixed model used included a fixed-effect term of time (T1, T2, T4) and a random participant intercept. β coefficient estimates represent the average changes in plasma sodium concentration at the post-HD timepoints (T2 and T4) relative to the pre-HD baseline (T1) in the ‘Time-Series’ study. R^2^_m_ and R^2^_c_ represent the proportion of variance explained by solely the fixed effect and by the full model, respectively.

Abbreviations: T1, pre-HD1; T2, post-HD1; T4, pre-HD2; R^2^_m_, marginal R^2^; R^2^_c_, conditional R^2^.

### Effects of an acute intradialytic cycling bout on intradialytic changes in calf muscle and plasma sodium concentrations

**Figure 6:**
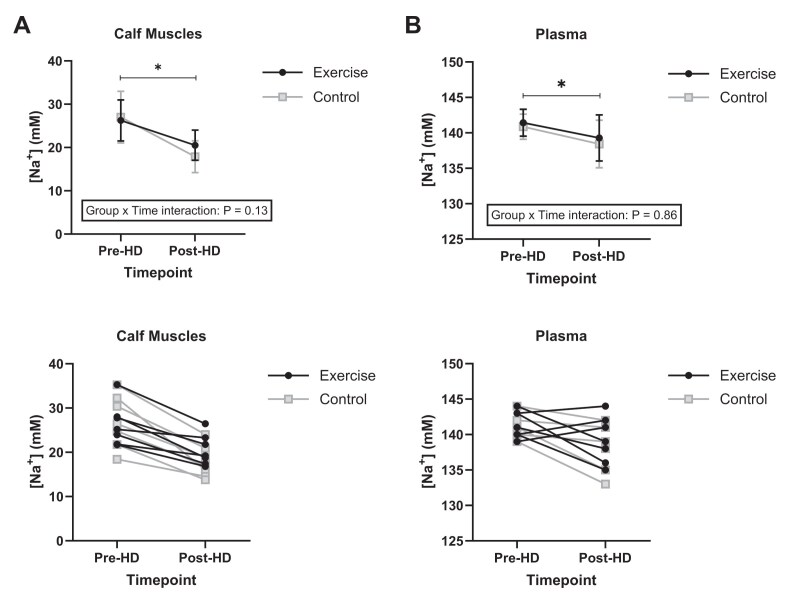
Intradialytic sodium concentration changes in (A) calf muscles and (B) plasma during the control and exercise HD sessions. Summary and individual data (*n* = 7) are shown in top and bottom insets respectively. Summary data are presented as mean ± SD. Linear mixed model analysis showed that both calf muscle and plasma sodium concentrations were reduced from pre- to post-HD (*P < .05), where the reductions did not differ between control and exercise HD (i.e. nonsignificant Group × Time interaction). HD, hemodialysis; [Na^+^], sodium concentration.

Figure 6 and Table 8 together show that both calf muscle and plasma sodium concentrations decreased from pre- to post-HD, with these decreases being non-statistically different between control and exercise HD sessions ([control vs. exercise]: muscle, −9.15 ± 3.84 vs. −5.72 ± 2.82 mM, *P* = .13; plasma, −2.43 ± 2.88 vs. −2.14 ± 3.76 mM, *P* = .86). Additionally, for both calf muscle and plasma sodium concentrations, no differences were found between control and exercise HD regardless of timepoints, as indicated by non-significant group effects (muscle, *P* = .40; plasma, *P* = .37).

**Table 8:** Comparisons of intradialytic changes in calf muscle and plasma sodium concentrations between control and exercise HD sessions (linear mixed model analysis).

	Control HD(*n* = 7)			Exercise HD(*n* = 7)			Linear mixed model analysis^a^			
							*P* value			
	Pre-HD^b^	Post-HD^c^	∆post-pre (%change)	Pre-HD^d^	Post-HD^e^	∆post-pre (%change)	Group effect	Time effect	Group × Time effect	R^2^_m_/R^2^_c_
Calf muscle sodium concentration, mM	27.0 ± 6.0	17.9 ± 3.7	−9.15(−33.8%)	26.3 ± 4.7	20.5 ± 3.5	−5.72(−21.8%)	.40	<.001	.13	0.46/0.75
Plasma sodium concentration, mM	140.9 ± 1.8	138.4 ± 3.4	−2.43(−1.7%)	141.4 ± 1.9	139.3 ± 3.3	−2.14(−1.5%)	.37	.009	.86	0.20/0.43

Values are shown as mean ± SD. R^2^ _m_ and R^2^_c_ represent the proportion of variance explained by solely the fixed effect and by the full model, respectively. R^2^_m_, marginal R^2^; R^2^_c_, conditional R^2^.

a The linear mixed model used included fixed-effect terms for group (control vs. exercise), time (pre-HD vs. post-HD), the group × time interaction, and a random participant intercept.

b Timepoint T1 in the ‘Time-Series’ study.

c Timepoint T2 in the ‘Time-Series’ study.

d Timepoint T5 in the ‘Na-Exercise’ study.

e Timepoint T6 in the ‘Na-Exercise’ study.

## DISCUSSION

This observational study used ²³Na-MRI to characterize calf muscle sodium kinetics in MHD patients during and between HD sessions. In the ‘Time-Series’ study, calf muscle sodium concentration decreased during HD, returned to baseline by 24 h post-HD, and was numerically lower than baseline before the subsequent HD session. In the ‘Na-Exercise’ study, an acute 30-min intradialytic cycling bout did not exert a statistically significant effect on the intradialytic reduction in muscle sodium concentration compared with control HD. Plasma sodium kinetics followed a similar pattern as that observed for muscle sodium.

The novel and principal finding of this study is the rebound of calf muscle sodium concentration at 24 h post–HD, followed by a potential partial reduction before the next HD session, as assessed by calf ²³Na–MRI. Consistent with prior reports by Dahlmann *et al*. [16] and Kopp *et al*. [17], we confirmed an immediate post-HD reduction in calf muscle sodium concentration. Importantly, for the first time, we observed a complete return to pre–HD levels at 24 h post–HD, followed by a trend toward partial reduction immediately before the subsequent HD session (Fig. 2 and Table 5). We also found that the observed muscle sodium kinetics may not be primarily explained by intra- or interdialytic fluctuations in fluid volume parameters (TBW, ECF, ICF) or body weight, as suggested by the linear mixed–model adjustments for these variables (Table 5). This latter observation aligns with MRI findings by Prestwich *et al*. [26], who reported no correlation between intradialytic reductions in muscle sodium concentration and concomitant decreases in muscle hydration. Collectively, the muscle sodium kinetics observed in our ‘Time–Series’ study suggest a complex equilibrium mechanism governing body sodium during and between HD sessions. This mechanism appears to diverge from that regulating fluid volumes, supporting the existence of non–osmotic sodium storage or redistribution. Our finding of post-HD muscle sodium kinetics also underscores the importance of standardizing the timing of ²³Na–MRI relative to HD sessions, particularly when using repeated MRI to evaluate the effects of an intervention on muscle sodium concentration in MHD patients.

In the ‘Time-Series’ study, after the complete rebound at 24 h post-HD, calf muscle sodium concentration again tended to decrease before the next HD session (Table 5; ∆T4 − T1: −2.85 mM, *P* = .07), though this decline was smaller than the immediate post-HD reduction (Table 5; ∆T2 − T1: −9.15 mM, *P* < .001). To assess whether this potential post-rebound decrease was related to diuretics use, we performed a linear mixed-model analysis including diuretics as a covariate (Table 6). In the non-diuretic subgroup, the kinetic pattern resembled that of the overall cohort, with the reduction immediately before the subsequent HD session reaching statistical significance (Table 6; ∆T4 − T1: −4.64 mM, *P* = .01). This suggests that the post-rebound decline in muscle sodium concentration may not be attributable to diuretic use or residual diuresis. Interestingly, although the overall time-course trajectories did not differ significantly by diuretic use (subgroup × time interaction: *P* = .12), we observed a trend toward a larger rebound at 24 h post-HD in the diuretics subgroup compared with the non-diuretics subgroup (∆T3 − T1_Diuretics_ − ∆T3 − T1_Non-Diuretics _= 5.33 mM, *P* = .05). While these findings are preliminary rather than definitive due to the small cohort, they suggest a potential divergence in post-HD tissue sodium redistribution between diuretic-treated MHD patients and those not receiving diuretics. Further investigation is warranted to confirm this observation and to determine whether other clinically relevant subgroups (e.g. diabetes status, BMI categories, residual kidney function) demonstrate differing post-HD muscle sodium kinetics.

In the ‘Na-Exercise’ study, we expected a larger immediate decrease in calf muscle sodium concentration during the exercise HD session, given prior evidence that intradialytic cycling can enhance solute clearance [18,19,21]. However, the immediate pre- to post-HD reduction did not differ significantly between sessions and was numerically smaller during the exercise session (control vs. exercise: −9.15 vs. −5.72 mM; Fig. 6 and Table 8). Several factors may explain this. First, the study was powered only to detect an immediate pre- to post-HD decrease in muscle sodium concentration, limiting our ability to detect an additional exercise effect. Second, our 30-min cycling bout was shorter than in prior studies [18–21] and may have been insufficient to influence intradialytic muscle sodium changes, despite using an intensity consistent with protocols that enhance phosphate clearance [27]. Third, exercise timing may have influenced the outcome. Prior work suggests that exercise later in HD may better augment solute clearance when muscle perfusion becomes rate-limiting [22], whereas our protocol involved cycling during the first hour. Finally, ^23^Na-MRI studies in healthy individuals show acute post-exercise increases in muscle sodium concentration, likely reflecting sarcolemmal sodium influxes and edema [28,29]. This aligns with our finding of a numerically smaller decrease in muscle sodium concentration during the exercise session, suggesting that an exercise–induced increase may have partially offset the intradialytic reduction. Although inconclusive, these results help guide future work on how intradialytic exercise may influence muscle sodium kinetics in MHD patients.

Plasma sodium kinetics during and between HD was studied and compared to that of muscle sodium in this study. As seen in Fig. 5, plasma sodium concentration decreased during HD and fully recovered before the next HD session, largely resembling the observed rebound kinetics of muscle sodium (Fig. 2) when omitting the muscle sodium measurements at T3. Furthermore, the decrease in plasma sodium concentration during HD was not affected by the intradialytic cycling bout, which is similar to our findings for muscle sodium concentration (Fig. 6). The resemblance between changes in plasma and muscle sodium concentration appears reasonable, as intradialytic tissue sodium reduction is secondary to HD-enforced intravascular sodium removal [16]. However, our repeated measures correlation analysis indicated that the intradialytic sodium concentration change (∆[Na^+^]) in calf muscle was not correlated with the ∆[Na^+^] in plasma (r_rm_ = −0.41, 95% CI: −0.86, 0.42; *P* = .32; Supplemental Fig. S1). This finding may suggest that the buffering between muscle and plasma sodium may be too complex to be captured by simple correlation analysis.

The two primary mechanisms for dialytic sodium removal are convection and diffusion [30,31]. Dialysis parameters such as ultrafiltration volume (UFV) and pre-HD dialysate-to-plasma sodium gradient (d-pNa) have therefore been considered potential determinants of intradialytic muscle sodium removal and long-term muscle sodium storage. However, in this study neither UFV nor d-pNa correlated with intradialytic changes in calf muscle sodium concentration (Supplemental Fig. S2) or with pre-HD calf muscle sodium concentration (Supplemental Fig. S3). Our findings, together with prior ^23^Na-MRI studies [16,32,33], indicate that HD acutely reduces muscle sodium concentrations. The magnitude of this acute reduction, as well as its longer-term influence on muscle sodium levels, does not appear to be predictable from dialysis parameters such as UFV or d-pNa.

Although skin sodium concentrations were quantified, these data are presented as supplementary because the 6.36–mm voxel size of ^23^Na–MRI may limit measurement precision in the thin skin layer. In the ‘Time Series’ study, skin sodium showed a significant overall time effect across two consecutive HD sessions (*P* = .02; Supplemental Table S1 and Supplemental Fig. S4), with concentrations decreasing immediately post–HD (−3.94 mM, *P* < .01), remaining numerically below baseline at 24 h (−2.03 mM, *P* = .14), and falling significantly below baseline before the subsequent session (−3.77 mM, *P* < .01). This latter reduction was unlikely to be driven by diuretics use (Supplemental Table S2). Notably, the overall time effect became non–significant after adjustment for total body water (*P* = .09; Supplemental Table S1), indicating that volume changes may contribute to the observed skin sodium kinetics. In the ‘Na–Exercise’ study, skin sodium concentration did not significantly decrease during either the exercise or control HD session (Supplemental Table S3 and Supplemental Fig. S5), in contrast to muscle sodium. Overall, these findings should be interpreted cautiously but suggest both parallels and distinctions between skin and muscle sodium kinetics during intra– and interdialytic intervals.

This study has several important limitations. First, the small sample size was powered only to detect a decrease in calf muscle sodium concentration during HD. Second, the two-component studies were not randomized, which may have introduced bias. Third, the patient characteristics warrant consideration. Despite taking an average of four antihypertensive medications, participants had mean pre-HD and post-HD SBP/DBP values above 160/95 mmHg and 150/85 mmHg, respectively (Table 3). These values exceed the 2005 KDOQI blood pressure targets [34] and indicate inadequate blood pressure control. The relatively large UFV of ∼3 l, together with a plasma sodium concentration of about 141 mmol/l, a dialysate sodium concentration of 136 mmol/l, and an HD duration of roughly 3.83 h, suggests that dialytic sodium removal may have approached 600 mmol per session. This amount is considerably higher than the ∼350 mmol reported by Dahlmann *et al*. [16]. When combined with pre-HD ECF/TBW ratios (Table 3) above the 0.40 threshold for volume overload [35,36], these findings indicate that our cohort was likely fluid and sodium overloaded. Participants also had a high BMI of about 36 kg/m², which likely reflected excess adiposity rather than increased muscle mass based on the ^1^H MRI anatomy (Fig. 3B). Despite these differences, the pre-HD calf muscle sodium concentrations observed in this study (20–35 mmol/l; Table 6A) were consistent with values reported in age-matched MHD patients [16], which supports partial generalizability. A fourth limitation is the absence of data required for sodium mass quantification, including sodium content in spent dialysate, urinary sodium excretion, and dietary intake. As a result, our analysis focused on muscle sodium concentration kinetics rather than whole body sodium balance. Finally, in the ‘Na Exercise’ study, ^23^Na MRI was performed only immediately before and after the exercise HD session, which prevented comparison of post-HD muscle sodium rebound between the control and exercise conditions. Addressing these issues will be important for future studies that aim to clarify mechanisms regulating muscle sodium flux in MHD.

In conclusion, this study is the first to characterize post-HD muscle sodium rebound kinetics in MHD patients. Calf muscle sodium concentration decreased substantially during HD, returned to baseline within 24 h post–HD, and showed a possible partial decline again before the subsequent HD session. Notably, the intradialytic decrease in muscle sodium concentration was not clearly influenced by a single bout of intradialytic cycling and did not correlate with intradialytic changes in plasma sodium. Our findings support ^23^Na–MRI as a promising non–invasive tool for assessing tissue sodium. Larger studies are needed to confirm these results and guide muscle sodium management in the MHD population.

## Supplementary Material

sfag193_Supplemental_File

## Data Availability

The data underlying this article will be shared on reasonable request to the corresponding author.
