# Supplementary material for: The impact of intradialytic cycling on temporal changes in muscle sodium during hemodialysis
Source: Clin Kidney J. 2026 Jul 15;19(7):sfag193. doi: 10.1093/ckj/sfag193 (PMC13386503; doi:10.1093/ckj/sfag193)
Supplement: sfag193_Supplemental_File [file sfag193_supplemental_file.docx]

**SUPPLEMENTAL MATERIAL**

**Table of Contents**

[Supplemental Table 1. Changes (∆) in skin sodium concentration over time between 2 consecutive HD sessions (linear mixed model analysis) 2](#_Toc223874690)

[Supplemental Table 2. Changes (∆) in skin sodium concentration over time between 2 consecutive HD sessions, adjusted for diuretics use subgroups (linear mixed model analysis) 4](#_Toc223874691)

[Supplemental Table 3. Comparison of intradialytic changes in skin sodium concentration between control and exercise HD sessions (linear mixed model analysis) 5](#_Toc223874692)

[Supplemental Figure 1. Repeated-measures correlations between the pre-to-post HD sodium concentration changes (∆[Na^+^]) in calf muscle and the ∆[Na^+^] in plasma (n = 7). 6](#_Toc223874693)

[Supplemental Figure 2. Repeated-measures correlations between the pre-to-post HD sodium concentration changes (∆[Na^+^]) in calf muscle and (A) ultrafiltration volume and (B) pre-HD dialysate-to-plasma sodium gradient (n = 7). 7](#_Toc223874694)

[Supplemental Figure 3. Repeated-measures correlations between the pre-HD calf muscle sodium concentration ([Na^+^]) and (A) ultrafiltration volume and (B) pre-HD dialysate-to-plasma sodium gradient (n = 7). 8](#_Toc223874695)

[Supplemental Figure 4. Skin sodium concentrations at each timepoint between 2 consecutive HD sessions. 9](#_Toc223874696)

[Supplemental Figure 5. Intradialytic skin sodium concentration changes during the control and exercise HD sessions. 10](#_Toc223874697)

# **Supplemental Table 1. Changes (∆) in skin sodium concentration over time between 2 consecutive HD sessions (linear mixed model analysis)**

| Skin sodium concentration, mM (n = 7) | | | | | | | | | |
| --- | --- | --- | --- | --- | --- | --- | --- | --- | --- |
|  | **∆T2 − T1** | | **∆T3 − T1** | | **∆T4 − T1** | | **Time effect**  **P value** | **R^2^_m_/ R^2^_c_** | **AIC** |
| Model | **β**  **(95% CI)** | **P value** | **β**  **(95% CI)** | **P value** | **Β**  **(95% CI)** | **P value** |  |  |  |
| Model 1  (Time) | −3.94  (−6.66, −1.21) | < 0.01 | −2.03  (−4.76, 0.69) | 0.14 | −3.77  (−6.49, −1.04) | < 0.01 | 0.02 | 0.03/0.92 | 169.5 |
| Model 2  (Time + TBW) | −3.26  (−6.18, −0.28) | 0.03 | −1.98  (−4.69, 0.73) | 0.15 | −3.38  (−6.15, −0.57) | 0.02 | 0.09 | 0.09/0.92 | 170.2 |
| Model 3  (Time + ECF) | −3.69  (−6.38, −0.97) | 0.01 | −2.21  (−4.91, 0.48) | 0.11 | −3.85  (−6.53, −1.17) | < 0.01 | 0.03 | 0.08/0.92 | 169.5 |
| Model 4  (Time + ICF) | −3.85  (−6.76, −0.89) | 0.01 | −2.01  (−4.75, 0.74) | 0.15 | −3.71  (−6.51, −0.88) | 0.01 | 0.04 | 0.03/0.92 | 171.5 |
| Model 5  (Time + BW) | −4.23  (−7.01, −1.45) | < 0.01 | −2.21  (−4.95, 0.53) | 0.11 | −3.88  (−6.59, −1.16) | < 0.01 | 0.02 | 0.12/0.93 | 170.8 |

Skin sodium concentrations measured in the “Time-Series” study were analyzed with the following linear mixed models:

Model 1: the model included a fixed-effect term of time (T1 – T4) and a random participant intercept.

Model 2: Model 1 plus volume of total body water (TBW) as a fixed-effect term.

Model 3: Model 1 plus volume of extracellular fluid (ECF) as a fixed-effect term.

Model 4: Model 1 plus volume of intracellular fluid (ICF) as a fixed-effect term.

Model 5: Model 1 plus body weight (BW) as a fixed-effect term.

β coefficient estimates represent the average changes in skin sodium concentration at the post-HD timepoints (T2, T3, and T4) relative to the pre-HD baseline (T1) in the “Time-Series” study. R^2^_m_ and R^2^_c_ represent the proportion of variance explained by solely the fixed effect and by the full model, respectively. AIC is a model selection criterion, with a lower value indicating a better model fit.

Abbreviations: T1, pre-HD1; T2, post-HD1; T3, 24 hours post-HD1; T4, pre-HD2; R^2^_m_, marginal R^2^; R^2^_c_, conditional R^2^; AIC, Akaike information criterion.

# **Supplemental Table 2. Changes (∆) in skin sodium concentration over time between 2 consecutive HD sessions, adjusted for diuretics use subgroups (linear mixed model analysis)**

|  | β Estimate (95% CI) | P value |
| --- | --- | --- |
| Time |  | 0.01 |
| T2 vs. T1 | −2.99 (−6.25, 0.27) | 0.07 |
| T3 vs. T1 | −2.95 (−6.21, 0.31) | 0.08 |
| T4 vs. T1 | −4.77 (−8.03, −1.50) | < 0.01 |
| Subgroup |  | 0.25 |
| Diuretic vs. Non-diuretic | 6.93 (−6.80, 20.7) | 0.29 |
| Time × subgroup interaction |  | 0.23 |
| T2 × Diuretic | −2.21 (−7.20, 2.77) | 0.37 |
| T3 × Diuretic | 2.14 (−2.83, 7.13) | 0.39 |
| T4 × Diuretic | 2.33 (−2.65, 7.31) | 0.35 |

Skin sodium concentrations measured in the “Time-Series” study were analyzed using a linear mixed-effects model that included fixed effects for time (T1–T4), subgroup (diuretics use vs. non-use), the time × subgroup interaction, and a random intercept for each participant.

The β estimates for time represent the mean change in skin sodium concentration at post-HD time points (T2, T3, and T4) relative to the pre-HD baseline (T1) within the non-diuretic subgroup.

The β estimate for subgroup represents the mean difference in skin sodium concentration between the diuretic and non-diuretic subgroups at T1.

The β estimates for the time × subgroup interaction represent the additional change in skin sodium concentration at each post-HD time point relative to T1 in the diuretic subgroup compared with the corresponding change in the non-diuretic subgroup (i.e., ∆T_i_ − T_1_*_Diuretic_* - ∆T_i_ − T_1_*_Non-Diuretic_*; i = 2, 3, 4).

Abbreviations: T1, pre-HD1; T2, post-HD1; T3, 24 hours post-HD1; T4, pre-HD2.

|  | Control HD  (n = 7) | | | Exercise HD  (n = 7) | | | Linear mixed model analysis^a^ | | | |
| --- | --- | --- | --- | --- | --- | --- | --- | --- | --- | --- |
|  | **Pre-HD**^b^ | **Post-HD**^c^ | **∆post-pre**  **(%change)** | **Pre-HD**^d^ | **Post-HD**^e^ | **∆post-pre**  **(%change)** | **P value** | | | **R^2^_m_/ R^2^_c_** |
|  |  |  |  |  |  |  | **Group effect** | **Time effect** | **Group x Time effect** |  |
| Skin sodium concentration, mM | 20.2 ± 10.1 | 16.3 ± 7.9 | −3.94  (−19.5%) | 17.7 ±12.0 | 16.4 ± 7.4 | −1.30  (−7.4%) | 0.41 | 0.09 | 0.38 | 0.03/0.81 |

# **Supplemental Table 3. Comparison of intradialytic changes in skin sodium concentration between control and exercise HD sessions (linear mixed model analysis)**

Values are shown as mean ± SD. R^2^_m_ and R^2^_c_ represent the proportion of variance explained by solely the fixed effect and by the full model, respectively. R^2^_m_, marginal R^2^; R^2^_c_, conditional R^2^.

^a^The linear mixed model used included fixed-effect terms for group (control vs. exercise), time (pre-HD vs. post-HD), the group × time interaction, and a random participant intercept.

^b^Timepoint T1 in the “Time-Series” study.

^c^Timepoint T2 in the “Time-Series” study.

^d^Timepoint T5 in the “Na-Exercise” study.

^e^Timepoint T6 in the “Na-Exercise” study.

# **Supplemental Figure 1. Repeated-measures correlations between the pre-to-post HD sodium concentration changes (∆[Na^+^]) in calf muscle and the ∆[Na^+^] in plasma (n = 7).**

**
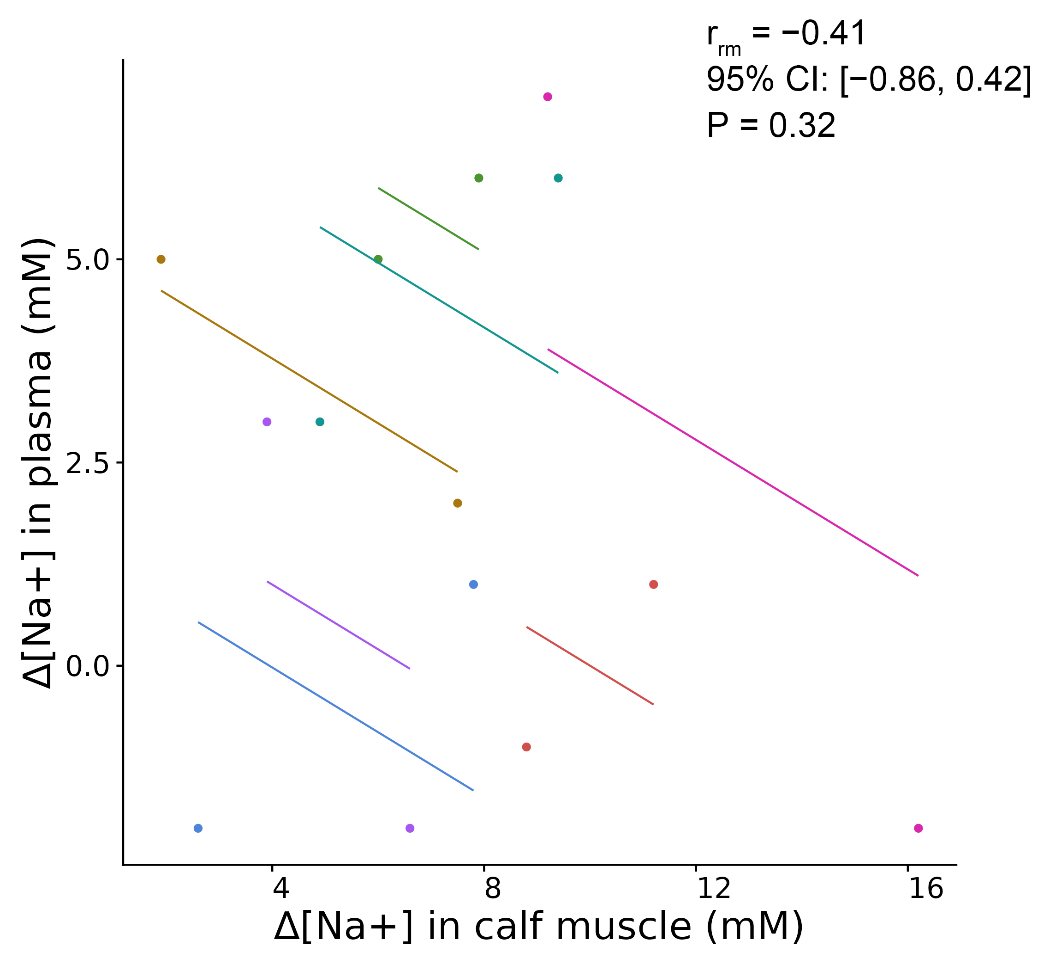
**

Intradialytic removal of calf muscle sodium is not associated with the removal of plasma sodium. Each pair of data points in the same color denotes the observations from the same patient obtained during the control and exercise HD sessions. Solid lines in corresponding colors represent the repeated measures correlation fitted for each patient. r_rm_, repeated-measures correlation coefficient.

# **Supplemental Figure 2. Repeated-measures correlations between the pre-to-post HD sodium concentration changes (∆[Na^+^]) in calf muscle and (A) ultrafiltration volume and (B) pre-HD dialysate-to-plasma sodium gradient (n = 7).**

**
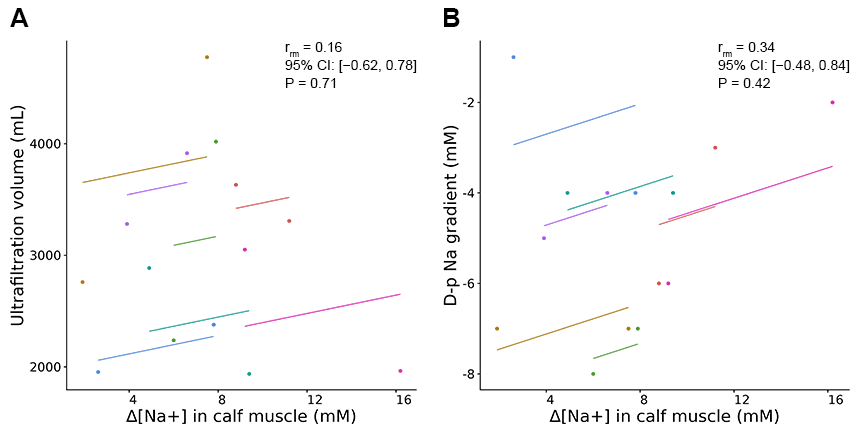
**

Intradialytic removal of calf muscle sodium is associated neither with the ultrafiltration volume nor with the pre-dialysis dialysate-to-plasma sodium gradient. Each pair of data points in the same color denotes the observations from the same patient obtained during the control and exercise HD sessions. Solid lines in corresponding colors represent the repeated measures correlation fitted for each patient. D-p Na gradient, pre-dialysis dialysate-to-plasma sodium gradient; r_rm_, repeated-measures correlation coefficient.

# **Supplemental Figure 3. Repeated-measures correlations between the pre-HD calf muscle sodium concentration ([Na^+^]) and (A) ultrafiltration volume and (B) pre-HD dialysate-to-plasma sodium gradient (n = 7).**

**
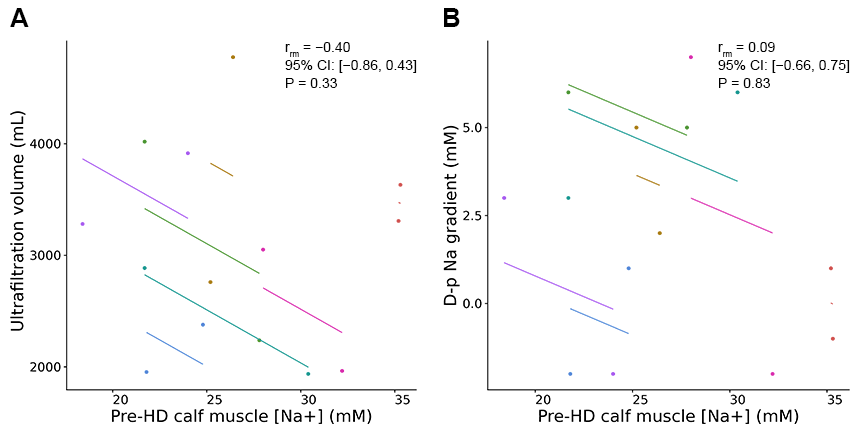
**

Pre-HD calf muscle sodium concentration is associated neither with the ultrafiltration volume nor with the pre-dialysis dialysate-to-plasma sodium gradient. Each pair of data points in the same color denotes the observations from the same patient obtained during the control and exercise HD sessions. Solid lines in corresponding colors represent the repeated measures correlation fitted for each patient. D-p Na gradient, pre-dialysis dialysate-to-plasma sodium gradient; r_rm_, repeated-measures correlation coefficient.

# **Supplemental Figure 4. Skin sodium concentrations at each timepoint between 2 consecutive HD sessions.**

**
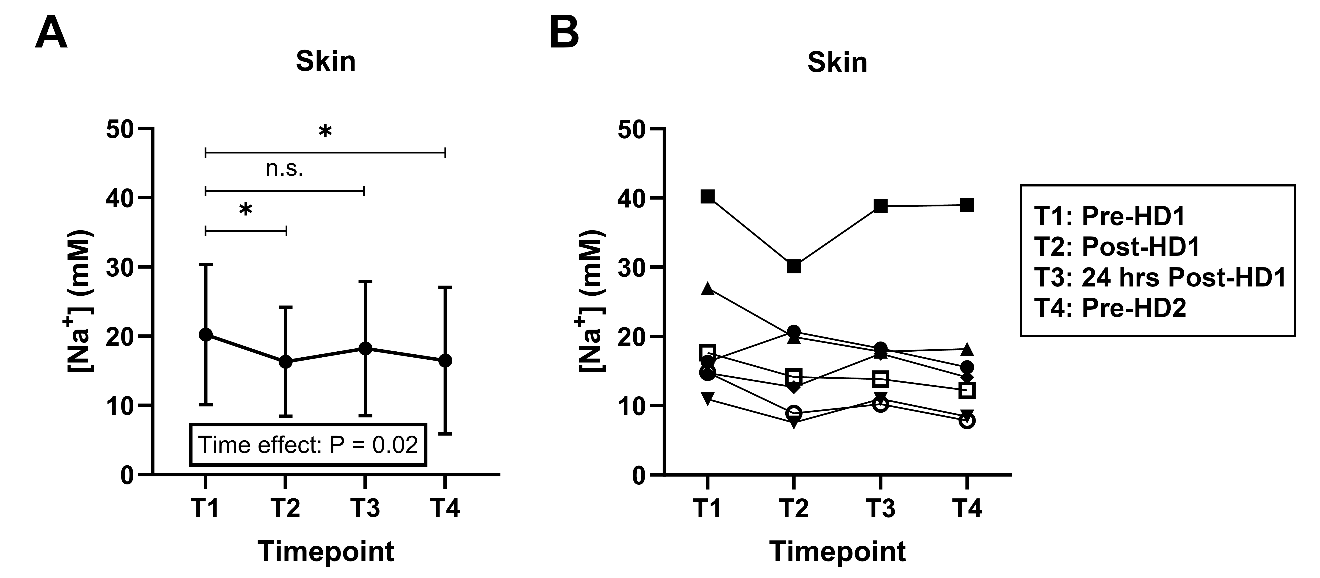
**

(A) Summary data. (B) Individual data (n = 7). The summary data plot displays the means and SDs of the skin sodium concentrations at timepoints T1 – T4: T1, 20.2 ± 10.1 mM; T2, 16.3 ± 7.9 mM; T3, 18.2 ± 9.7 mM; T4, 16.5 ± 10.6 mM. Linear mixed model analysis showed a significant time effect for skin sodium concentrations over T1 – T4 (P = 0.02). The analysis further showed that, compared with T1, skin sodium concentration decreased at T2, remained non-significantly lower at T3, and decreased further at T4. ^*^P < 0.01; n.s., non-significant. HD, hemodialysis; [Na^+^], sodium concentration.

# **Supplemental Figure 5. Intradialytic skin sodium concentration changes during the control and exercise HD sessions.**


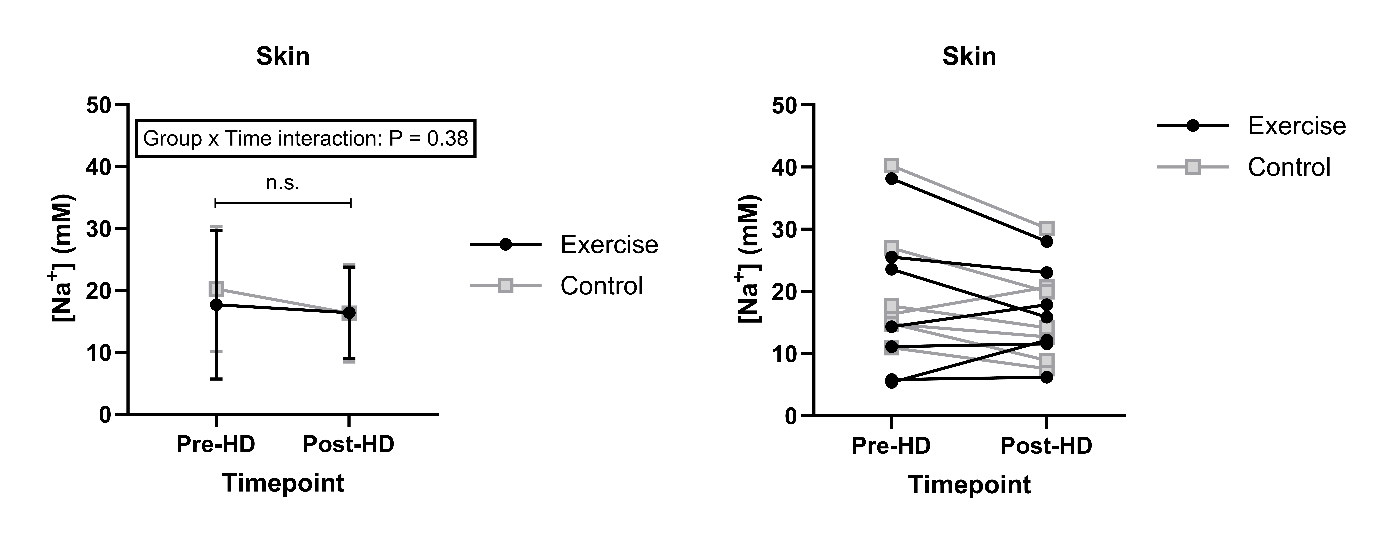


Summary and individual data (n = 7) are shown in left and right insets respectively. Summary data are presented as mean ± SD. Linear mixed‑effects analysis showed that skin sodium concentration did not change significantly from pre‑HD to post‑HD in either the exercise or control HD sessions (i.e., nonsignificant Group × Time interaction and Time effect). HD, hemodialysis; [Na^+^], sodium concentration; n.s., nonsignificant.
